# Supplementary material for: Fingerprinting and chemotyping approaches reveal a wide genetic and metabolic diversity among wild hops (Humulus lupulus L.)
Source: PLoS One. 2025 May 6;20(5):e0322330. doi: 10.1371/journal.pone.0322330 (PMC12054859; doi:10.1371/journal.pone.0322330)
Supplement: S1 Fig — Identified peaks are annotated from 1 to 12 according to S2 Table. NL: Normalization Level, FTMS: Fourier Transform Mass Spectrometry, ESI: Electrospray Ionisation. (XCalibur software - Qual Browser application, Thermo Fisher Scientific). (DOCX) [file pone.0322330.s001.docx]

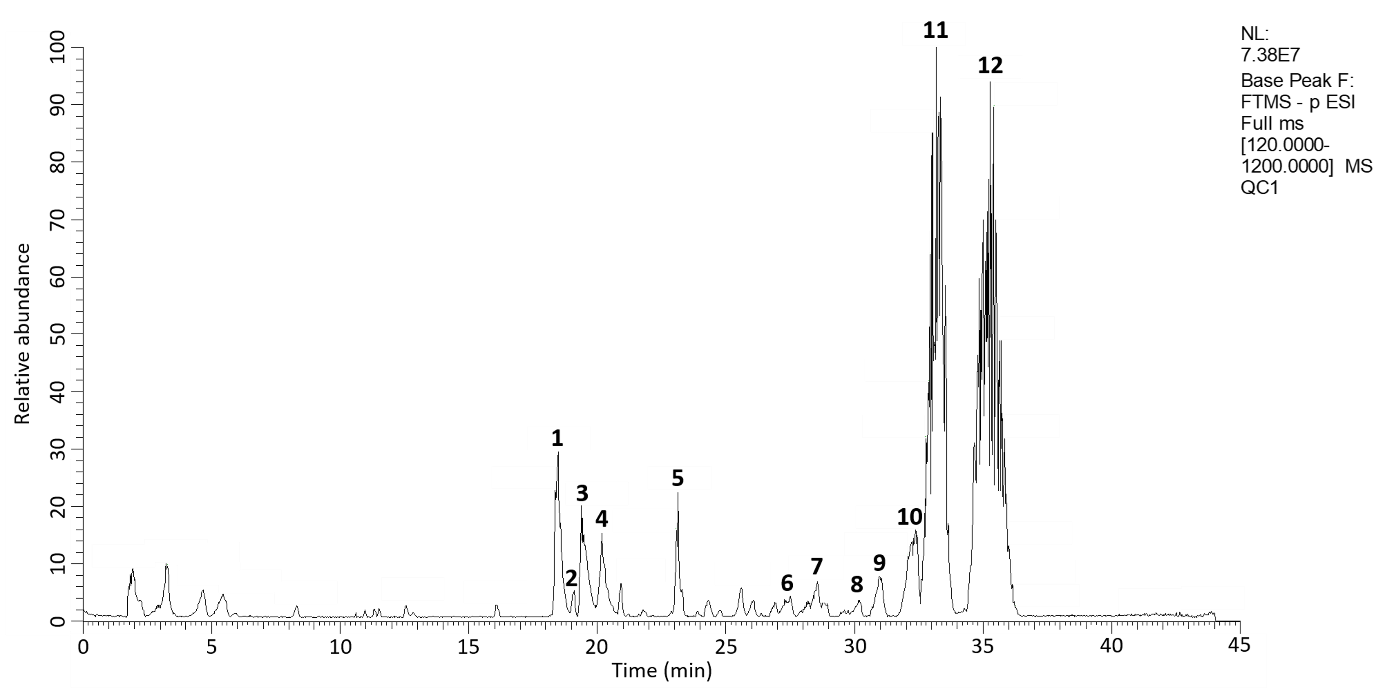


**S1 Fig. UHPLC-MS chromatogram ([M-H]^-^) from Quality Control (QC) of hop leaf extract.** Identified peaks are annotated from 1 to 12 according to S2 Table. NL: Normalization Level, FTMS: Fourier Transform Mass Spectrometry, ESI: Electrospray Ionisation. (XCalibur software - Qual Browser application, Thermo Fisher Scientific).
