# Supplementary material for: Fingerprinting and chemotyping approaches reveal a wide genetic and metabolic diversity among wild hops (Humulus lupulus L.)
Source: PLoS One. 2025 May 6;20(5):e0322330. doi: 10.1371/journal.pone.0322330 (PMC12054859; doi:10.1371/journal.pone.0322330)
Supplement: S2 Fig — The resulting dendrogram was made using the “Manhattan” distance, and “ward.D2” clustering method. (DOCX) [file pone.0322330.s002.docx]

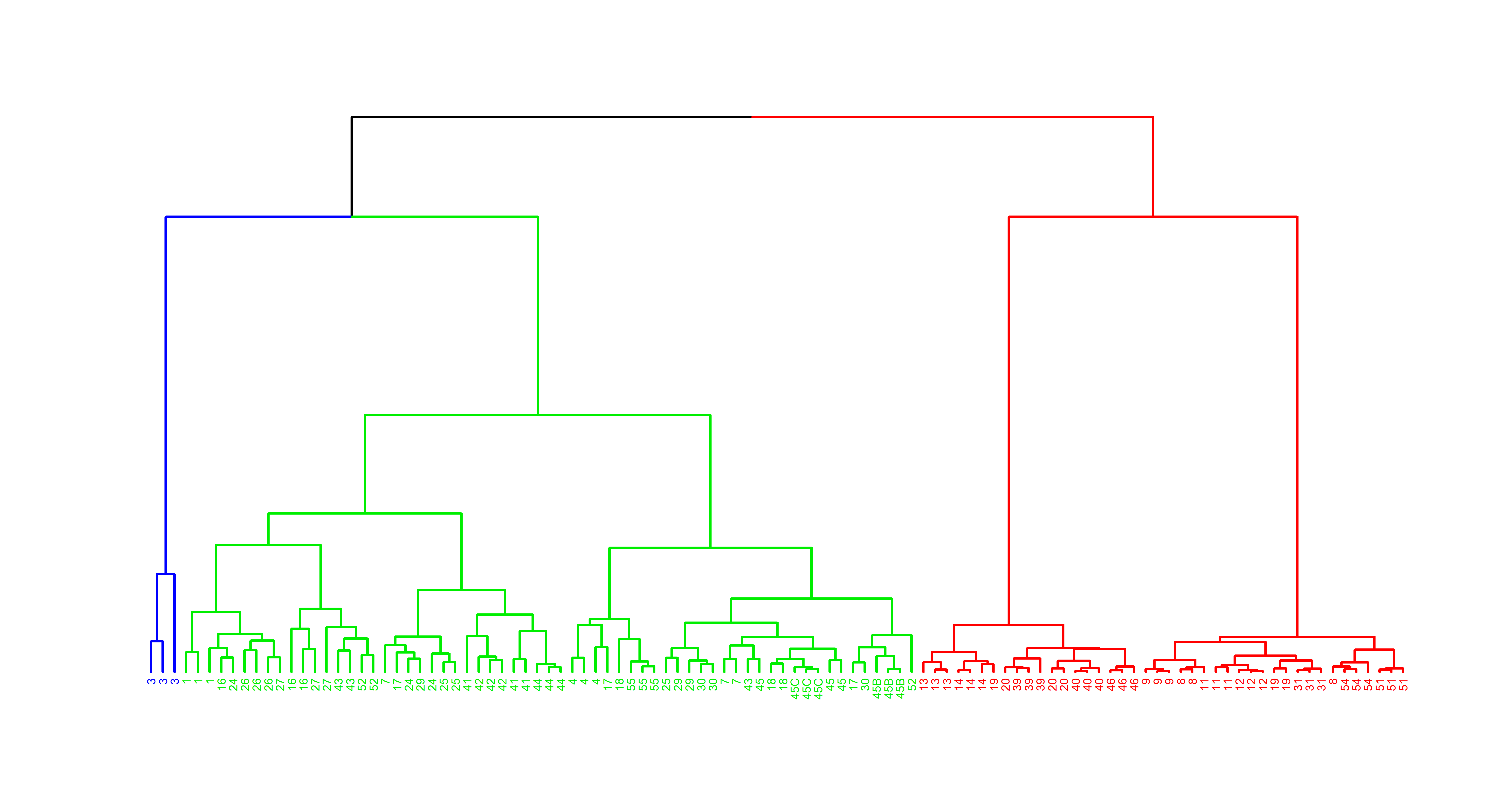


**S2 Fig. Hierarchical Ascending Classification (HAC) analysis based on hops leaf metabolic content.** The resulting dendrogram was made using the “Manhattan” distance, and “ward.D2” clustering method.
